# qSOFA should replace SIRS as the screening tool for sepsis

**DOI:** 10.1186/s13054-016-1562-4

**Published:** 2016-12-28

**Authors:** Stefano Franchini, Andrea Duca

**Affiliations:** Emergency Department, Ospedale San Raffaele Scientific Institute, Via Olgettina 60, Milan, 20132 Italy

Vincent JL, Martin GS and Levy MM recently wrote an article in Critical Care entitled “qSOFA does not replace SIRS in the *definition* of sepsis” [1]. In this paper they specified that “the qSOFA is meant to be used to raise suspicion of sepsis and prompt further action but it is not a replacement for SIRS and is not part of the definition of sepsis”.

One of the starting points that induced the Sepsis-3 consensus taskforce to set out in search of better entry criteria than the systemic inflammatory response syndrome (SIRS) criteria was precisely that SIRS criteria perform poorly on both “discriminant validity” and “convergent validity”[2]. In order to accomplish their task, they identified patients with suspected infection among 1.3 million health record cases and, after comparing the performance of several different clinical criteria, they came out with the quick sequential organ failure assessment (qSOFA) score, whose predictive validity for in-hospital mortality outside the ICU was statistically better than SIRS [3].

The fact that nonspecific SIRS criteria will “generally” continue to aid in the identification and diagnosis of infection was repeatedly affirmed in the Sepsis-3 consensus article [2]. Besides, when the SIRS criteria were first proposed as a screening tool for sepsis [4], they were meant to be applied to patients with “suspected infection”, just as the qSOFA is intended to be used now. However, while the SIRS criteria were essentially based only on expert-consensus [4], the qSOFA criteria were identified through large multivariate statistics and confirmatory analyses, where they proved to perform better than the SIRS criteria [3].

The qSOFA was derived and conceived on the basis of retrospective data, and thus, from now on, the clinical research should and will work hard to prospectively validate the soundness of this tool, in terms of its screening capacity. However, based at least on the currently available evidence, we believe that, although qSOFA does not replace SIRS in the *definition* of sepsis, it should indeed replace SIRS as *the screening tool* for sepsis.

We would like to know if Vincent and colleagues agree with this assumption, and we would also like to ask them if, after the Sepsis-3 consensus definitions, the SIRS criteria still retain a real operative role in the process of *defining* and/or *screening* sepsis or if they could be, at least operatively, dismissed.

## Abbreviations

qSOFA: quick sequential organ failure assessment
SIRS: systemic inflammatory response syndrome
