# Supplementary material for: qSOFA should replace SIRS as the screening tool for sepsis
Source: Crit Care. 2016 Dec 28;20:409. doi: 10.1186/s13054-016-1562-4 (PMC5192569; doi:10.1186/s13054-016-1562-4)
Supplement: Related texts

# Reply: Authors’ response

**Authors:** Jean-Louis Vincent, Greg S. Martin, Mitchell M. Levy

We thank Drs Franchini and Duca for their comments. Physicians have long used fever, associated tachycardia and altered white blood cell count as signs of infection … we have never needed the SIRS criteria to help with this and we don’t need the qSOFA for this either. Furthermore, qSOFA does not replace SIRS as a screening tool for sepsis because it was conceived, derived and validated as a prognostic tool. Moreover, sepsis is more often identified from associated unexplained organ dysfunction than from infection [5].

The use of qSOFA as an alarm signal should be further validated, keeping in mind that it is not specific for sepsis. Patients with many other conditions, including severe heart failure, blood loss, pulmonary embolism and any form of acute circulatory failure (shock), can have hypotension, altered mental status and hyperventilation, thus meeting the qSOFA criteria without having sepsis. But, it is still important to identify these patients and act quickly, whatever the underlying cause. The best screening tools for sepsis remain within the minds of clinicians, suspecting infection and assessing organ function using an array of criteria that so far have eluded complete description.
